# Identifying languages in a novel dataset: ASMR-whispered speech

**DOI:** 10.3389/fnins.2023.1120311

**Published:** 2023-06-15

**Authors:** Meishu Song, Zijiang Yang, Emilia Parada-Cabaleiro, Xin Jing, Yoshiharu Yamamoto, Björn Schuller

**Affiliations:** ^1^Chair of Embedded Intelligence for Health Care and Wellbeing, University of Augsburg, Augsburg, Germany; ^2^Educational Physiology Laboratory, The University of Tokyo, Tokyo, Japan; ^3^Institute of Computational Perception, Johannes Kepler University Linz, Linz, Austria; ^4^Group on Language, Audio, & Music, Imperial College London, London, United Kingdom

**Keywords:** ASMR, dataset, CNN, LSTM, whispered speech

## Abstract

**Introduction:**

The Autonomous Sensory Meridian Response (ASMR) is a combination of sensory phenomena involving electrostatic-like tingling sensations, which emerge in response to certain stimuli. Despite the overwhelming popularity of ASMR in the social media, no open source databases on ASMR related stimuli are yet available, which makes this phenomenon mostly inaccessible to the research community; thus, almost completely unexplored. In this regard, we present the ASMR Whispered-Speech (ASMR-WS) database.

**Methods:**

ASWR-WS is a novel database on whispered speech, specifically tailored to promote the development of ASMR-like unvoiced Language Identification (unvoiced-LID) systems. The ASMR-WS database encompasses 38 videos-for a total duration of 10 h and 36 min-and includes seven target languages (Chinese, English, French, Italian, Japanese, Korean, and Spanish). Along with the database, we present baseline results for unvoiced-LID on the ASMR-WS database.

**Results:**

Our best results on the seven-class problem, based on segments of 2s length, and on a CNN classifier and MFCC acoustic features, achieved 85.74% of unweighted average recall and 90.83% of accuracy.

**Discussion:**

For future work, we would like to focus more deeply on the duration of speech samples, as we see varied results with the combinations applied herein. To enable further research in this area, the ASMR-WS database, as well as the partitioning considered in the presented baseline, is made accessible to the research community.

## 1. Introduction

The Autonomous Sensory Meridian Response (ASMR) is a physical response triggered by sensory stimuli—often described as “tingles”—which typically start in the scalp before spreading in waves across the body (Gallagher, 2016). Although the perception of ASMR content has shown to be subjective (Smith and Snider, 2019), varying across individuals, a typical ASMR brings a pleasurable sense of calm (Gallagher, 2016). Due to this, ASMR has been recently considered in a variety of initiatives aimed to promote wellness, such as meditation (Barratt and Davis, 2015), therapy (Gallagher, 2016), and specific processes aimed at reducing stress (Barratt and Davis, 2015). Research on ASMR is, however, still reasonably new (Barratt and Davis, 2015; Fredborg et al., 2017), finding a surge in attention due to the spread of online content—shared predominately through YouTube—intended to evoke a relaxing sensation (Andersen, 2015). Although ASMR can be triggered by audio-visual and tactile stimuli, the auditory component of ASMR related content is essential. Indeed, audio stimuli such as whispered-speech, rustling paper, tapping of fingers, or crinkling plastic, are typical scenarios used by users with the intention of being relaxed (Andersen, 2015).

The development of a system for ASMR content understanding is of critical importance due to the growing popularity of ASMR as a potential means of promoting relaxation, reducing stress, and improving overall wellbeing (Andersen, 2015). However, the subjective and heterogenous nature of ASMR triggers make it difficult to measure and quantify, and there is a lack of standardized methods for identifying and categorizing ASMR videos. Language Identification (LID) serves as the initial step in building an ASMR content understanding system by facilitating the automatic classification of videos according to their language, which is a crucial determinant of their content and target audience (Mehrabani and Hansen, 2011; Monteiro et al., 2019). For example, ASMR videos in English may exhibit distinct triggers or styles from those in other languages, and understanding these variances can aid in personalizing and optimizing the ASMR experience for viewers. Furthermore, the presence of whispered speech (Bartz et al., 2017) in ASMR videos poses challenges for traditional speech processing techniques. LID can enable the differentiation of whispered speech in various languages, thereby enabling more accurate analysis and comprehension of ASMR content. Thus, the development of a system for ASMR content understanding that incorporates LID as a foundational element is integral to advancing our knowledge and comprehension of ASMR and its potential benefits.

With the aim of filling the gap between LID systems and speech-based ASMR, we present, to the best of our knowledge, the first multi-language database specially designed to investigate ASMR from whispered-speech in a variety of languages. To encourage the development of further ASMR-based whispered-speech language identification systems—being of interest for both the ASMR and speech processing communities—a baseline aimed to identify the language in ASMR whisper-speech, resulting from the application of Convolutional Neural Network (CNN) and Long Short-Term Memory (LSTM) architectures on acoustic features—Mel-Frequency Cepstral Coefficients (MFCCs) and Logarithmic Mel-Spectrogram (logMel), is also presented.

## 2. Related work

The rise of online communities specifically created to elicit ASMR “tingles” (Fredborg et al., 2017), evidences an always increasing interest in ASMR. There are several factors that distinguish ASMR from other atypical sensory experiences, such as “frisson”, the sudden tingling sensations that occurs also during an emotional response to music (Fredborg et al., 2017). For instance, although both, ASMR and frisson, present an affective component and tend to occur while a given individual is mindful and fully engaged with the triggering stimulus, the tingles associated with frisson tend to spread rapidly throughout the body, whereas those related to ASMR may last up to several minutes (Del Campo and Kehle, 2016). Some research into the neural substrates linked to ASMR confirmed that sensorial stimuli, such as light touch, can bring an internal sensation of deep relaxation (Lochte et al., 2018). Indeed, recent research has demonstrated that ASMR are related to the activation of specific brain regions associated to the sensation of pleasure (Lochte et al., 2018). Showing also, that individuals who experience ASMR, present a greater default mode network functional connectivity (Raichle, 2015). Current research suggested that auditory stimuli—particularly whispered speech—are crucial in experiencing ASMR (Poerio et al., 2018).

Whispered speech, also known as unvoiced speech and typically produced with no vocal-cord vibration, is characterized by low-energy (Zhou et al., 2019). As opposed to “normal” speech, the speech produced through the use of voiced sounds with harmonic excitation, whispered speech is produced with broad-band noise (Zhou et al., 2019), being, for instance, the typical form of communication for individuals diagnosed with *aphonia* (Zhou et al., 2019). In our hypersonic world, whispered speech, which usually requires closeness between speaker and listener (Li, 2011), presents an inherent affective component. Indeed, from the ASMR enthusiasts prospective, it has been described as a recreation of maternal intimacy (Cheadle, 2012). In recent years, different machine learning tasks related to whispering have emerged, such as whispered speech recognition (Xueqin et al., 2016), whispered emotional speech recognition (Deng et al., 2016), and whisper to normal speech conversion (Pascual et al., 2018); yet, despite the gained attention of this research topic and the variety of available whisper datasets (Silva et al., 2016), ASMR-specific whispered-speech datasets have not yet been developed.

## 3. ASMR-WS database: description

The ASMR-WS (Autonomous Sensory Meridian Response Whispered-Speech) database is made up of 38 WAV audio clips (mean length 17 min, std dev 37 min, and a total duration of 10 h and 36 min) retrieved from YouTube along with language labels. Their purpose was to evoke ASMR. The database contains unvoiced speech produced by 38 adult female speakers in seven languages (Chinese, English, French, Italian, Japanese, Korean, and Spanish). For each language, at least four speakers were considered, and except for Chinese (whose content lasts 37 min), all the other languages present audio content longer than 1 h (for the number of speakers and content length of each language cf. Table 1).

**Table 1:** Speaker information for each language in the ASMR-WS database.

**Languages**	**Duration**	**#**	**Mean**	**Std**
Chinese	37 min	5	7.4	4.1
English	1 h 40 min	5	20.0	5.2
French	2 h 50 min	8	21.0	1.6
Italian	1 h 10 min	5	14.0	2.6
Japanese	1 h 27 min	7	12.4	3.1
Korean	1 h 28 min	4	22.0	3.6
Spanish	1 h 24 min	4	21.0	5.6

Reporting total duration, number (#) of speakers, and the mean and standard deviation (std) for duration (in minutes) that each speaker appears.

### 3.1. Data selection, acquisition, and validation

ASMR related content from YouTube is presented in an audio-visual form; yet, considering that auditory stimuli are crucial for ASMR, we decided to keep only audio. In order to increase the probability of finding speech in a given language, in the results' filter from the YouTube search, proximity to the location of major cities was prioritized. Following this criterion, seven languages were selected: Chinese, English, French, Italian, Japanese, Korean, and Spanish, those with a sufficient amount of clips. To retrieve suitable ASMR related content in unvoiced speaking style, obvious keywords such as ‘ASMR' and “whisper”, as well as others related to these, e.g., “reading a book”, were considered (translated into the targeted languages) to appropriately filter the YouTube results.

The YouTube videos were retrieved through the Application Programming Interface, which allows developers to retrieve information from YouTube's database. The audio layer was subsequently extracted in WAV format encoded in single channel 16 kHz 16 bit PCM. Only content with at least 120 s of whisper speech, the minimum considered to enable the recognition of the target language, was taken into account. In addition, audio content presenting background music/noise or recorded at low quality, as well as that with more than one speaker, was dismissed. Considering that for some languages there were no male speaker, in order not to collect a heavily gender-imbalanced database, only female speech was taken into account. Importantly, only content associated to a Creative Commons license was take into account.^1^ All these criteria were applied subjectively by two auditors (authors of the presented work), and only samples targeted as valid by both auditors were considered part of the database.

## 4. Experimental setup

A critical challenge for automatic language identification is to achieve superior classification performance in the context of the shortest possible speech segments (Van Segbroeck et al., 2015). To this end, previous works have shown that the success in performing this task on speech segments with the length of 1 s or even shorter, leads to rapid language identification for inference (Van Segbroeck et al., 2015). In this work, we conducted experiments to evaluate our database, taking into account three different segment lengths: (0.5, 1, and 2 s) An automatic language identification model takes the acoustic feature sets, such as logMel and MFCCs, that are extracted from each segment and predicts its belonging language type.

### 4.1. Data partition and truncation

For the experiments, the database was split into training, development, and test sets, as appears in Table 2 under column “Speakers”. The partitioning assures a participant-independent setting. The primary rationale for utilizing a participant-independent setting is to mitigate potential sources of bias in the analysis process by circumventing the effects of individual variation in data interpretation (Luo et al., 2018). Further, the audio recordings are truncated with 50% overlap (Charpentier and Stella, 1986) in length for each segment length, resulting in a total of 151,765 chunks of 0.5 s, 75,853 chunks of 1 s, and 37,898 chunks of 2 s, respectively. The reason why we applied overlap technique is to achieve more complete and continuous representations of the speech signal (Charpentier and Stella, 1986).

**Table 2:** Distribution of the database in train, (dev)elopment, and test sets with the number of speakers, and different segments.

**Languages**	**Speakers**			**0.5 s**			**1 s**			**2 s**		
	**Train**	**Dev**	**Test**	**Train**	**Dev**	**Test**	**Train**	**Dev**	**Test**	**Train**	**Dev**	**Test**
Chinese	3	1	1	6,057	1,030	1771	3,026	514	885	1,511	256	442
English	3	1	1	18,383	2,691	3,172	9,189	1,345	1,585	4,593	672	792
French	4	2	2	18,746	12,373	9,586	9,370	6,185	4,791	4,682	3,091	2,394
Italian	3	1	1	9,245	3,722	3,722	4,620	1,860	1,860	2,307	929	929
Japanese	3	2	2	10,313	6,306	4,171	5,237	3,152	2,085	2,616	1,574	1,042
Korean	2	1	1	15,015	2,976	2,650	7,506	1,487	1,324	3,751	743	661
Spanish	2	1	1	13,003	2,914	3,755	6,499	1,456	1,877	3,248	727	938
**Σ**	20	9	9	90,926	32,012	28,827	45,447	15,999	14,407	22,708	7,992	7,198

### 4.2. Feature sets

Two kinds of acoustic feature sets, logMel and MFCC, were extracted using the open-source openSMILE toolkit (Eyben et al., 2010), which has been widely applied for speech-, audio- and health-related tasks (Song et al., 2019; Yang et al., 2019; Han et al., 2020; Qian et al., 2020). Previous research has demonstrated that logMel and MFCC coefficients are effective in capturing pertinent information in speech signals, such as spectral characteristics and modulation patterns (Meghanani et al., 2021). Furthermore, more intricate feature sets may result in overfitting and computational inefficiency (Padi et al., 2021), which can adversely impact performance. Hence, the selection of a limited set of features, namely logMel and MFCC coefficients, was made to achieve optimal performance while ensuring computational efficiency.

#### 4.2.1. LogMel feature set

LogMel frequency is a representation of the Logarithmic Mel-scale on the short-time frequency, successfully applied in a range of acoustic tasks, such as speech feature enhancement and acoustic scene classification, amongst others (Ren et al., 2019, 2020). The advantage of applying the logarithmic Mel-scale, on one side, is an easy implementation with higher resolution in the time-frequency domain (Farooq and Datta, 2002), on the other, the low complexity of its estimation algorithm (Ambikairajah et al., 2011), which reduces the computational cost. In this study, we use 26-band logMel, and the first and second delta regression coefficients (Eyben et al., 2010). The Delta coefficients are extracted based on the logMel frequencies in each audio segment with a length of 0.01 s.

#### 4.2.2. MFCC feature set

MFCCs are a representation derived from logMEL frequencies by computing the cepstrum of the melodic frequencies. MFCCs are one of the most commonly used filterbank-based parameterization methods for speech processing applications, such as speech recognition, speaker verification/identification, and language identification (Eyben et al., 2013). We gain the advantage of low dimensionality and independence of the corruption across feature dimensions (Acero et al., 2006). In this work, we extract 39-dimensional MFCC features, including 13 MFCC coefficients, the first and the second delta regression coefficients, in which both delta coefficients have 13 dimensions.

### 4.3. Classification models

Recently, deep learning models have been successfully applied to the tasks of language modeling (Sagha et al., 2016). Previous experience with LSTM on this topic showed good results on short segments for a limited number of languages (Gelly and Gauvain, 2017). Another popular network for this use case are CNNs, which has been explored in language identification in order to obtain an utterance level representation (Wang et al., 2019). For these reasons, in our experiments, we utilize LSTM and CNNs for baseline results.

The LSTM model contains a single layer to model the sequential input, and the output of the last hidden unit is mapped to the number of classes through a sequence of dense layers. The number of neurons of each dense layer are 64, 128, 256, 127, and 64. The architecture of our CNN model contains two convolutional layers, activated by a ReLU function. Both convolutional layers use the kernel size of (5, 5) and stride size of (1, 1). Max pooling is applied for each layer with the kernel size of (2, 2). The output feature maps of the second convolutional layer is flattened, and then projected to the number of classes via a dense layer. Softmax is used to normalize the model output. Other hyper-parameters used in this work to train both models, LSTM and CNN, are given in Table 3.

**Table 3:** LSTM and CNN training hyperparameters.

**Parameter**	**Value**
Optimizer	Adam
Learning rate	0.001
Activation function	ReLu
Batch-size	128
Train epochs	100
Loss function	Cross-entropy

## 5. Baseline results

We presented the performance of our LSTM and CNN models for different feature representations and different segment lengths in Table 4. Besides classification accuracy (Acc), Unweighted Average Recall (UAR) is used in this work to evaluate the LID performance, as it is commonly used for unbalanced multi-class classification tasks, for example, in the Native Language Identification Sub-Challenge held within the INTERSPEECH 2016 Computational Paralinguistics Challenge (Schuller et al., 2016).

**Table 4:** The performances [(Acc)uracy [%] and UAR [%]] of the proposed LSTM and CNN models on different durations.

		**0.5 s**				**1 s**				**2 s**			
		**MFCC**		**logMel**		**MFCC**		**logMel**		**MFCC**		**logMel**	
		**Dev**	**Test**	**Dev**	**Test**	**Dev**	**Test**	**Dev**	**Test**	**Dev**	**Test**	**Dev**	**Test**
LSTM	Acc [%]	76.77	54.52	86.23	75.45	77.92	77.73	84.31	65.03	74.17	70.63	87.04	82.63
	UAR [%]	64.64	62.63	70.12	64.15	68.15	70.09	68.84	54.06	54.81	58.68	71.39	62.36
CNN	Acc [%]	56.25	52.68	55.15	54.53	86.16	88.81	83.57	89.49	88.00	**90.83**	73.23	77.39
	UAR [%]	68.85	59.72	67.61	62.63	78.84	83.12	65.68	83.86	80.76	85.74	53.40	70.49

In each duration, the performances of MFCC and logMel features are presented on the (dev)lopment and test set. Bold value indicates the best result.

**Figure 1:**
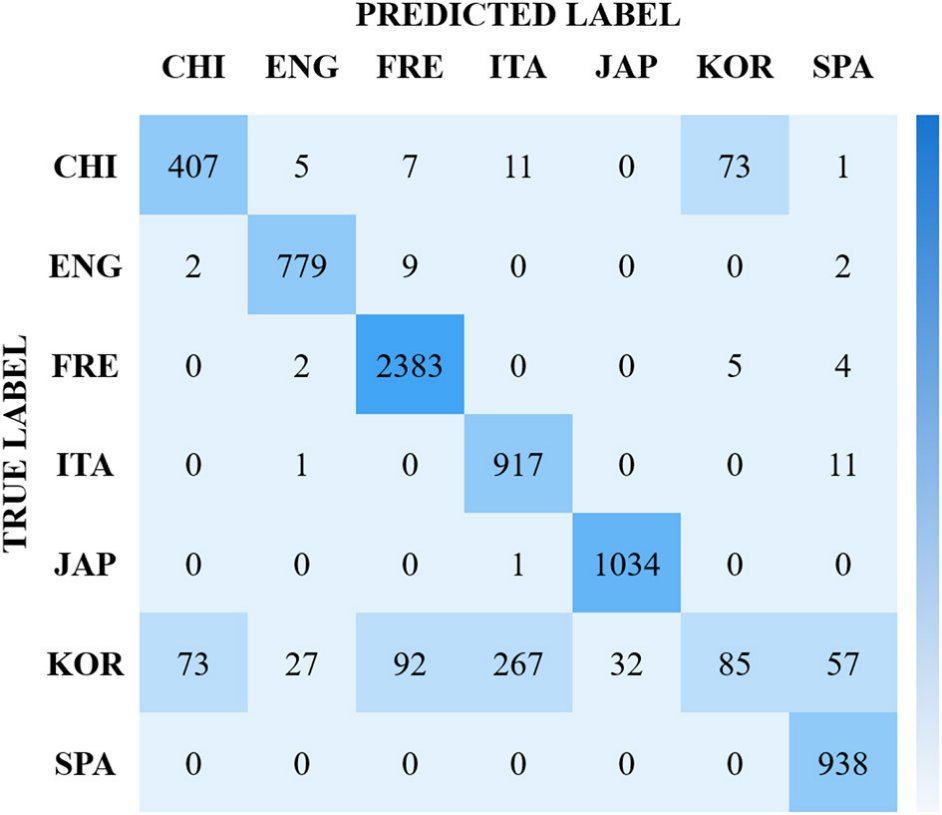
Confusion matrix for the best baseline results of the ASMR-WS dataset. Reporting 90.83% accuracy on test, using 2 s samples, in a CNN/MFCC paradigm.

From observing our result, we see that the best identification results for the 7-class language task comes from CNNs utilizing MFCCs with at best 90.83 % accuracy. We see from the confusion matrix in Figure 1 that the French language is identified better than all other languages, with Korean being confused most. We speculate that this confusion may come from linguistic similarities, such as phonology and prosody (Madhu et al., 2017).

For example, although the roman languages considered, French, Italian, and Spanish, present lexical and grammatical similarities, their acoustic elements differ (Parada-Cabaleiro et al., 2018, 2019), which may have been the cause for increased classification accuracy. In this case, Korean (where we see our highest confusion), the languages presents phonologically short and long vowels, and the length of which is not represented orthographically (Ingram and Park, 1997). This complexity in phonetic structure may be the reason for an impaired classification result, and suggests to us that these prosodic elements play a strong role in this task.

We also notice that for shortness of utterances results, for 0.5 s identification tasks, LSTM perform better than CNN for both MFCC and logMel features. We argue that LSTM stores more temporal state of data than CNN. For logMel features within the CNN classifier, the 1 s task works better than the 0.5 second and 2 s tasks. Changing from 0.5 to 1 s, the performance of the CNN classifier for logMel improves tremendously. For 2 s identification tasks, the CNN classifier for MFCC feature works very satisfyingly. We speculate that the reason may stem from the ability of CNNs to “grab” details at a specific node are better than LSTM due to grid-like topology.

### 5.1. Limitations

Although our baseline results confirmed that the ASMR-WS database is promising for ASMR speech research, there are some places which could be improved: (i) A standard LSTM network only predicts the labels based on the past time stamps in a forward direction. Bidirectional LSTM extends the single-directional LSTM network by introducing an additional backward direction (Cai et al., 2019) if non-causality is an option. We will optimize our classifier architecture as bidirectional LSTM to improve the performance. (ii) Except machine learning models used in this work, an i-vector model has shown promising to extract effective representations for speech recognition tasks (Song et al., 2013). Therefore, i-vector model based features will be extracted for the task of unvoiced LID in the future. (iii) Another current limitation of the database is that Chinese content is still below 1 h of length. In this regards, we plan to collect Chinese ASMR speech from Chinese social media to balance the content of this language. (iv) Additionally, it is important to acknowledge that our dataset presents a gender imbalance, as a significant proportion of ASMR material available on the YouTube platform is generated by female speakers. Such gender domination can give rise to two primary concerns: firstly, certain gender-specific ASMR triggers may be more attractive to individuals of a particular gender, and secondly, the underrepresentation of male gender can potentially compromise the generalizability of the models. Nonetheless, establishing a dataset that encompasses a balanced representation of both male and female speakers is currently a challenging task. In order to overcome this limitation, we plan to amass more ASMR data from male speakers in the future. (v) Our results for language identification of 2-s audio snippets showed promising performance for the seven languages considered in our study. However, we acknowledge that the identification rate for Roman languages, such as French and Italian, may be affected by the shorter snippet duration. Therefore, it would be interesting to investigate the detection rate of different languages at various snippet durations, including 0.5 and 1 s, to better understand the impact of duration on language identification. It is worth noting that different languages may exhibit varying optimal audio lengths, and investigating this aspect could be a potential direction for further research in the field of ASMR content understanding.

## 6. Conclusions

In this study we outline and present baselines for the first of its kind ASMR-WS dataset, which includes seven languages from 38 female speakers. In order to establish a benchmark for the dataset we perform a series of language identification tasks and developed two state-of-the-art architectures, namely LSTM- and CNN-based, processing three duration's of speech samples. Our experiments have shown promising results for the dataset, as well as for the task of whisper-based language identification. Of note, we find that an accuracy of up 90.83% is possible for the 7-class task. For future work, we would like to focus more deeply on the duration of speech samples, as we see varied results with the combinations applied herein. The same would apply to the use of acoustics features. in which it may be of interest to explore other well-known speech dataset including low-level descriptors from the well-known openSMILE toolkit. For whispered speech language identification specifically, it would be of interest to explore more closely the results we obtained from the Korean language, as developing a model which focuses on this seemingly more complex language, may prove fruitful. Lastly, through the use of state of the art audio-based architectures, it may be of interest to apply the ASMR-WS dataset to other novel tasks, including ASMR activity detection and ASMR whispered speech generation.

## Data availability statement

The raw data supporting the conclusions of this article will be made available by the authors, without undue reservation.

## Author contributions

MS and ZY are responsible for data collection and analysis. EP-C, YY, and BS are responsible for paper rephrasing and proofreading. All authors contributed to the article and approved the submitted version.

## Footnotes

^1^The Creative Commons, CC BY license gives permission that, so long as the work is attributed, anyone can: adapt – remix, transform, and build upon the material for any purpose. The content validation was manually performed.
